# Protein clustering for cell polarity: Par-3 as a paradigm

**DOI:** 10.12688/f1000research.11976.1

**Published:** 2017-08-31

**Authors:** Tony J. C. Harris

**Affiliations:** 1 Department of Cell & Systems Biology, University of Toronto, Toronto, Canada

**Keywords:** Par-3 clusters, Protein clustering, cell polarity

## Abstract

The scaffold protein Par-3 ( *Drosophila* Bazooka) is a central organizer of cell polarity across animals. This review focuses on how the clustering of Par-3 contributes to cell polarity. It begins with the Par-3 homo-oligomerization mechanism and its regulation by Par-1 phosphorylation. The role of polarized cytoskeletal networks in distributing Par-3 clusters to one end of the cell is then discussed, as is the subsequent maintenance of polarized Par-3 clusters through hindered mobility and inhibition from the opposite pole. Finally, specific roles of Par-3 clusters are reviewed, including the bundling of microtubules, the cortical docking of centrosomes, the growth and positioning of cadherin–catenin clusters, and the inhibition of the Par-6–aPKC kinase cassette. Examples are drawn from *Drosophila, Caenorhabditis elegans*, mammalian cell culture, and biochemical studies.

## Introduction

**Figure 1.**
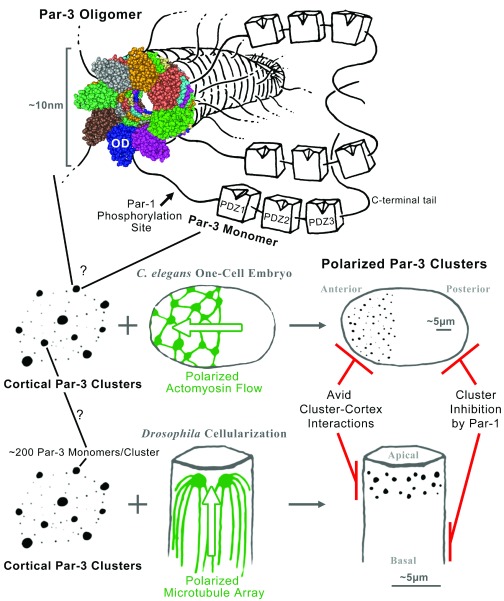
The organization and polarization of Par-3 clusters. The oligomerization domain of a Par-3 monomer mediates the formation of a helical fiber from which the linker regions and PDZ domains of Par-3 would emanate like branches of a tree. The space-filling structural model is a Cn3D view of the model of Zhang *et al*. (PDB: 3ZEE) ^14^. Somehow these fibers are organized into local Par-3 clusters scattered across the cell cortex. In the *Caenorhabditis elegans* one-cell embryo, a polarized actomyosin flow sweeps the Par-3 puncta to one pole, forming the anterior end of the embryo. In the cellularizing *Drosophila* embryo, a polarized microtubule array positions the Par-3 puncta to one end of each cell, forming the apicolateral domain. Once the polarity of Par-3 clusters is established, it can be maintained by avid interactions between the clusters and the cell cortex combined with inhibition of Par-3 complex formation at the opposite pole by Par-1 phosphorylation. See main text for further details.

As a scaffold protein central to the polarization of many animal cell types, Par-3 binds numerous molecules both for its recruitment to one pole of the cell and for downstream contributions to polarized cell function ^1– 3^. Par-3 contains many interaction sites, including an N-terminal oligomerization domain and three central PDZ domains, as well as binding motifs in its N- and C-terminal tails ( Figure 1). At the heart of Par-3 organization is Par-3 homo-oligomerization, clustering that is inhibited by Par-1, a kinase that typically localizes to the opposite pole of the cell. Additionally, Par-3 binds the Par-6–aPKC cassette to form the Par complex, although these players also function separately. Through these interactions and others, Par-3 helps polarize the cell cortex for the structure and function of cells and cell populations. For example, the polarization of epithelial cells into distinct apical, lateral, and basal cortices is essential for epithelia to act as selective barriers. Another significant example is cortical polarization for asymmetric cell division and cell type diversification.

This review focuses on the clustering of Par-3 and how this clustering contributes to cell polarity. Clustering provides a mesoscale organization important for molecules to transcend scales and impact cells. Many proteins cluster, or polymerize, for their roles in cells. For example, polarity proteins cluster to form plasma membrane landmarks in bacteria, yeast, and multicellular organisms ^4– 6^. Similarly, actin and microtubule polymers form networks that shape eukaryotic cells and form subcellular domains ^7, 8^, and cadherin–catenin clusters adhere cells together and organize multi-component cortical complexes ^9^. Similar to cytoskeletal and adhesion complexes, a Par-3 oligomerization mechanism has been defined structurally and is subject to regulation. Moreover, the polarization and effects of Par-3 clusters are intimately associated with cytoskeletal networks and adherens junctions. In this way, Par-3 clusters integrate with other mesoscale complexes to form large-scale networks for the structure and function of polarized cells.

## The Par-3 homo-oligomerization mechanism

In 2003, the labs of Shigeo Ohno and Daniel St Johnston independently reported the homo-oligomerization of the conserved N-terminal domain of Par-3, termed conserved region 1 (CR1) ^10, 11^. The Ohno lab noticed the co-localization and co-immunoprecipitation of distinct isoforms of mammalian Par-3 and discovered that these interactions required CR1. The sufficiency of CR1 for dimerization was demonstrated by the yeast two-hybrid protein interaction assay as well as by chemical cross-linking of purified CR1 into apparent dimers and additional higher-molecular-weight species. The St Johnston lab discovered a structural alignment of *Drosophila* CR1 with a bacterial protein that was known to oligomerize. The ability of the CR1 domain to dimerize was demonstrated by yeast two-hybrid assay, and interactions between larger portions of *Drosophila* Par-3, each including CR1, were shown for purified proteins and by co-immunoprecipitation from *Drosophila* extracts. Subsequently, CR1 of *Caenorhabditis elegans* Par-3 was also shown to dimerize ^12^.

Four years later, the lab of Mingjie Zhang published the structure of mammalian CR1 ^13^. For this structural determination, the group first confirmed homo-oligomerization of purified CR1 using gel filtration and chemical cross-linking assays that each revealed high-molecular-weight species expected for oligomers. High salt concentrations disrupted the oligomerization, implicating electrostatic interactions. Thus, charged residues were mutated and assayed for effects on oligomerization *in vitro*. Two mutated CR1 domains were pursued because they failed to oligomerize but seemed to maintain their individual structure: one converted an uncharged valine into a negatively charged aspartic acid residue (V13D), and the other converted a negatively charged aspartic acid into a positively charged lysine residue (D70K). With a monomeric form of CR1 in hand, the group pursued its 3D structure by NMR spectroscopy without the complications of oligomeric species. This CR1 structure, five β-strands forming a half β-barrel closed on its open side by two α-helices, was shown to be structurally similar to ubiquitin and PB1 domains, including those that mediate the interaction between Par-6 and aPKC. Strikingly, this structure had two negatively charged patches on one side and two positively charged patches on the other, implicating a head-to-tail oligomerization mechanism. This mechanism explained the loss of homo-oligomerization for the mutated domains with added or altered charges (V13D and D70K) and also predicted hetero-dimerization between the two mutated domains that was observed. Modeling of oligomerization predicted a helical filament with six units of CR1 per complete turn and a cross-sectional diameter of approximately 7 nm. By electron microscopy, CR1 was shown to form filaments with this diameter. Moreover, a larger fragment of Par-3, containing CR1 plus the three PDZ domains, formed filaments that were abolished with the manipulation of the charged residues of CR1. Subsequent analyses of the wild-type CR1 domain by crystallography, cryo-electron microscopy, and atomic force microscopy confirmed the head-to-tail oligomerization mechanism and showed that it mediated lateral interactions for 8.2 units of CR1 per turn in a left-handed, approximately 10 nm diameter helix involving additional interactions between each of its longitudinal layers ^14^ ( Figure 1). These data placed the Par-3 oligomerization mechanism on strong structural footing (hereafter CR1 is described as the Par-3 oligomerization domain).

Deletion of the Par-3 oligomerization domain in either mammalian or *Drosophila* model systems revealed its critical role in concentrating Par-3 at specific sites of the cell cortex, such as cell–cell junctions or non-junctional puncta, and in promoting Par-3 function in epithelial organization ^10, 11^. Two approaches revealed additionally that the homo-oligomerization of the domain, rather than any alternate interactions, was central to Par-3 localization in mammalian cell culture ^13^. First, both amino acid residue changes that disrupted oligomerization *in vitro* also abrogated cortical Par-3 concentration in cells. Second, replacement of the Par-3 oligomerization domain with oligomerization domains of other proteins conveyed effective Par-3 localization. These molecular approaches have revealed the importance of Par-3 oligomerization for complex formation inside cells, but the exact structure of these oligomers remains unknown *in vivo*.

Following these pioneering studies, further structure–function analyses revealed requirements for the oligomerization domain in other tissues and animals, but these same studies also identified contexts where the oligomerization domain was not essential for cortical accumulations ^12, 15, 16^. In such situations, molecular interactions with other domains and regions of Par-3 seem to suffice for Par-3 recruitment. Thus, Par-3 clustering can be based on Par-3 homo-oligomerization and/or Par-3 piggybacking on aggregates of interaction partners.

## The regulation of Par-3 homo-oligomerization

Approximately 60–70 amino acids downstream of the oligomerization domain, both mammalian and *Drosophila* Par-3 can be phosphorylated to create binding sites for 14-3-3 proteins ^17, 18^. For *Drosophila* Par-3, the kinase Par-1 catalyzes the phosphorylation ^17^, and for the mammalian Par-3, protein phosphatase 1 removes the phosphate group ^19^. Several pieces of data indicate that this phosphorylation, and 14-3-3 protein binding, inhibits Par-3 oligomerization. By yeast two-hybrid assay, an interaction was detected between the N-terminal 308 amino acid residues of *Drosophila* Par-3 (containing both the oligomerization domain and the Par-1 phosphorylation site but ending prior to the PDZ domains) and full-length Par-3, and this interaction was inhibited by the additional overexpression of 14-3-3ε ^17^. Implicating the Par-1 phosphorylation site, its conversion from serine to alanine eliminated the inhibitory effect of 14-3-3ε, and the authors assumed that yeast kinases phosphorylated the site in this assay ^17^. The same serine to alanine conversion led to mislocalization of Par-3 in clusters along the lateral membrane of *Drosophila* epithelial cells where Par-1 is enriched ^17^. Over-expression of mammalian Par-3 with the equivalent mutation disrupted the structure of epithelial cysts in culture, suggesting a dominant negative effect through interaction with endogenous Par-3 ^18^. However, the sequence between the oligomerization domain and the Par-1 phosphorylation site has no predicted structure and could thus extend a relatively long distance ( Figure 1). The structural basis for Par-3 oligomerization inhibition by 14-3-3 protein binding remains unclear.

Further evidence for the phospho-regulation of Par-3 oligomerization is confounded by two complexities. First, a distinct Par-1 phosphorylation site in the C-terminal end of Par-3 also recruits 14-3-3 proteins but inhibits interaction with aPKC ^17^. This C-terminal site seems to be the only one phosphorylated by Par-1 and bound by 14-3-3 proteins for *C. elegans* Par-3 ^17, 20^. Also, this second site plays a specific role in the polarization of *Drosophila* neuroblasts ^21^. However, for other assessments of Par-3 following perturbations of Par-1, phosphatases, or both phosphorylation sites, it is unclear whether responses are due to altered Par-3 oligomerization, to disrupted aPKC interactions, to both effects, or possibly to other effects ^19, 22– 24^. A second confounding issue is that the inhibition of Par-3 complexes by Par-1 and 14-3-3 proteins is often coupled with distinct, semi-redundant localization mechanisms ^17, 20, 22^.

## The polarization of Par-3 clusters across the cell

The regulated oligomerization of Par-3 offers a mechanism for forming molecular domains within the cell cortex. However, this mechanism may not be sufficient for establishing a large, single Par-3 domain capable of covering the anterior cortex of the one-cell *C. elegans* embryo, the apical cortex of a *Drosophila* neuroblast, or the apical circumference of an epithelial cell. Specifically, subunits with low valency form small chain-like clusters, rather than large-scale phase separations ^25^. Intriguingly, however, overexpression of the N-terminal 311 amino acid residues of Par-3 in the *Drosophila* oocyte produces large spherical aggregates of the construct in the center of the cell ^11^. The size and shape of these aggregates are hallmarks of phase separation ^25, 26^. Since the construct contains the oligomerization domain plus a substantial amount of additional sequence, other direct or indirect oligomerization sites might reside in this portion of Par-3 to convey higher valency interactions, although such sites have not been reported. This aggregation potential is not fulfilled, however, for the full-length protein which typically forms numerous foci at the cell cortex ( Figure 1). Thus, the addition of the three PDZ domains and the C-terminal tail restricts Par-3 to the cortex where its clustering is limited. Here, additional mechanisms are needed to polarize Par-3 clusters to one end of the cell.

For the whole-cell polarization of full-length Par-3, cytoskeletal networks have been shown to draw Par-3 clusters to one end of the cell ( Figure 1). In the one-cell *C. elegans* embryo, a whole-cell flow of cortical actomyosin draws Par-3 clusters together to form the anterior end of the embryo ^27^. In the cellularizing *Drosophila* embryo, a whole-cell microtubule array and the minus-end directed motor dynein draw Par-3 clusters to the apico-lateral domain next to centrosomes ^28^. In each case, the whole-cell cytoskeletal polarity arises independently of the Par-3 clusters, and in the absence of the cytoskeletal network, Par-3 clusters display wide dispersal over the full cell cortex.

The early *C. elegans* and *Drosophila* embryos differ in their initial plasma membrane identity, a difference that influences how Par-3 clusters are positioned. Prior to polarization of the *C. elegans* zygote, the plasma membrane is fully covered by Par-3 puncta and actomyosin networks, and Par-1 is cytosolic. Sperm entry induces the anterior flow of the actomyosin networks, and posterior loading of Par-1 then occurs ^29^. As the plasma membrane first forms in *Drosophila*, it is fully covered by Par-1 ^22^, and Par-3 clusters assemble in what would seem to be an inhibitory context. Providing an explanation for how such assembly occurs, Par-1 was shown to not only inhibit cortical Par-3 complex assembly but also promote Par-3 interactions with centrosomal microtubules ^30^. These interactions seem to promote Par-3 clustering, and the associations of Par-3 clusters and centrosomal microtubules are self-reinforcing ^30, 31^. This positive feedback can lead to extreme co-recruitment of Par-3 clusters and centrosomes into single, large cortical patches, and Par-1 inhibition by aPKC normally prevents this runaway assembly so that Par-3 clusters are distributed more evenly for the positioning of adherens junctions ^30, 31^. Interestingly, a self-reinforcing loop is also evident in the *C. elegans* embryo, as Par-3 promotes the actomyosin flow that displaces the Par-3 clusters ^27^. Whether this *C. elegans* loop has the same co-clustering potential as the *Drosophila* loop, and whether it is also counter-regulated, is unknown.

For both the *C. elegans* and the *Drosophila* embryos, the cytoskeletal networks responsible for polarizing Par-3 clusters are re-configured shortly thereafter. In *C. elegans*, the actomyosin re-configuration is associated with entry into the polarity maintenance phase as the cell begins mitosis ^27^, and in *Drosophila* aPKC initiates the down-regulation of centrosomal microtubules typical of epithelial cells ^31, 32^. As these large-scale cytoskeletal assemblies are diminished, other mechanisms become responsible for maintaining the established Par protein polarity ^33^. In *C. elegans*, individual Par proteins can diffuse across the equatorial boundary, but as whole populations they remain restricted to separate poles ^33^. One form of stabilization comes from the decreased mobility of Par-3 clusters ^34^. Further confinement of Par-3 is provided by Par-1 inhibition of Par-3 complex formation at the opposite pole ^34^. In fact, for *C. elegans* mutants lacking the actomyosin flow altogether, Par-3 can polarize anteriorly owing to posterior Par-1 inhibitory activity concentrated by a local microtubule array ^20^. Modeling of *C. elegans* polarization indicates that Par-3 polarity maintenance can indeed be explained by the persistence of anterior Par-3 clusters through avid cortical interaction and the repulsion of Par-3 from the posterior cortex by Par-1 ^35^. In *Drosophila*, analogous effects occur and a more molecularly robust framework has been discovered. As the Par-3 clusters are positioned, they additionally engage a network of circumferential cadherin–catenin clusters ^36^ and actin filaments organized by Canoe/Afadin ^37^, and multiple redundant cortical association mechanisms later arise ^15^. Just following the establishment of Par-3 cluster polarity, basolateral proteins such as Par-1, Lgl, and Dlg are displaced from the apical domain ^22, 38, 39^ and act basally to inhibit ectopic assembly of apical proteins ^22, 39– 41^. Thus, homo-oligomerization and engagement with other mesoscale structures seem to maintain Par-3 polarity in combination with inhibition from the opposite pole ( Figure 1).

Although Par-3 clusters have a higher-order organization that engages other complexes across the cell cortex, they have not been tied exclusively to any other network. For example, Par-3 clusters are important for positioning cadherin–catenin clusters in the early *Drosophila* embryo ^38, 42^, but the Par-3 clusters engage these adherens junction precursors with substoichiometry and distinct cluster dynamics ^36^. Moreover, the Par-3 clusters can form with major depletion of the cadherin–catenin clusters ^38^, and vice versa ^36^. A second example comes from the apical constriction of amnioserosa cells of the later *Drosophila* embryo. Here, Par-3 regulates the assembly–disassembly cycles of apical actomyosin networks ^43^. Par-3 clusters depend on myosin to be recruited to the apical domain ^44^ and coalesce in the apical domain with the assembly and contraction of an apical actomyosin network ^43^. However, the Par-3 clusters lack specific colocalization with the actomyosin networks, and when the networks periodically disassemble the Par-3 clusters disperse in the apical domain but retain their membrane association ^43^. In the one-cell *C. elegans* embryo, Par-3 clusters similarly lack clear colocalization with the actomyosin networks that polarize them but intermingle amongst the cables and foci of the networks ^27^. Par-3 cluster turnover rates may be slow enough for them to translocate across the cell as part of an advective flow driven by actomyosin, as shown for Par-6 ^45^ (also see Note added in proof).

## Specific roles of Par-3 clusters

Besides affecting the localization of Par-3, what effect does Par-3 clustering have? Assigning function to a specific pool of a protein is challenging. As discussed, compromising the oligomerization domain of Par-3 can lead to downstream effects but can also decrease the cortical localization of Par-3. Thus, it can be difficult to discern whether Par-3 clustering has a role beyond Par-3 localization.

One additional role of the Par-3 oligomerization domain has been identified biochemically ^46^. The oligomerization domain was shown to promote the bundling of microtubules by Par-3 *in vitro*. This effect was indirect. The homo-oligomerization of Par-3 out-competed an interaction between the C-terminal and N-terminal ends of Par-3 that otherwise inhibited a microtubule binding site involving the second and third PDZ domains of Par-3. Additionally, the homo-oligomerization seems to cross-link microtubule-bound Par-3 for the bundling of microtubules. These interactions were shown to affect the polarization and microtubule organization of cultured mammalian neurons.

Additionally, colocalization studies have implicated three activities of Par-3 clusters. First, Par-3 clusters closely colocalize with centrosomes during intestinal development in *C. elegans* ^47^, asymmetric male germline stem cell division in *Drosophila* ^48^, and without normal down-regulation of the positive feedback loop between Par-3 and centrosomal microtubules in the early *Drosophila* ectoderm ^30, 31^. These examples suggest Par-3 clusters aggregate to form cortical docking sites for centrosomes, sites that may involve local cortical dynein recruitment ^28, 31, 49^. Second, Par-3 clusters colocalize closely with cadherin–catenin clusters as they merge and grow in a Par-3-dependent way in the early *Drosophila* embryo ^36, 38, 50^. Thus, Par-3 clusters seem to act as scaffolds for the assembly of adherens junctions. Moreover, the Par-3 clusters seem to trap the cadherin–catenin clusters to position them around the apicolateral domain ^36^. This role in adherens junction positioning has also been linked to a Par-1-regulated shift in the position of both Par-3 clusters and cadherin–catenin clusters for *Drosophila* embryo epithelial folding ^24^. Finally, the association of Par-3 clusters with Par-6–aPKC may inhibit the adaptor-kinase cassette by sequestration. A non-phosphorylatable form of Par-3 makes the Par-3–Par-6–aPKC interaction highly stable and extreme co-clustering results ^51^. Moreover, sequences flanking the aPKC phosphorylation site of Par-3 can inhibit aPKC ^52^. During internalization of the *Drosophila* amnioserosa, apical Par-6–aPKC activity initially antagonizes actomyosin networks to promote their assembly–disassembly cycles ^43^, but aPKC-dependent accumulation of apical Par-3 leads to Par-3 clustering, colocalization with Par-6–aPKC, and a loss of actomyosin inhibition, three effects expedited by expressing the form of Par-3 with strengthened aPKC interaction ^44^.

## Concluding remarks

Since the discovery of Par-3 homo-oligomerization, much has been learned about Par-3 clusters, but many questions remain. For example, the exact structure of individual Par-3 clusters observed by light microscopy remains unclear, as does their relationship with the helical filaments formed by the oligomerization domain *in vitro*. Amino acid residues important for oligomerization *in vitro* impact Par-3 assembly *in vivo* ^12, 13^, and about 200 Par-3 monomers have been detected per cluster *in vivo* ^36^, but the arrangement of these monomers within a cluster remains unknown. Also, the structural basis for inhibiting Par-3 oligomerization by Par-1 phosphorylation and 14-3-3 protein binding remains ill-defined and potentially involves a relatively long-distance effect.

The higher-order organization of Par-3 clusters is also unclear. Par-3 has multiple sites for binding different lipid head groups ^53– 55^ and, together with homo-oligomerization, these interactions would be enough to explain the formation of a single cortical Par-3 cluster. There are three non-mutually exclusive organization mechanisms that could connect individual Par-3 clusters into larger networks: (1) the oligomerization of Par-3 could generate both clusters and extended filaments to interconnect the clusters; (2) a single dedicated network of non-Par-3 filaments could interconnect the Par-3 clusters; and/or (3) various networks could engage Par-3 clusters in a loose, non-dedicated, and potentially competitive way. No evidence exists for mechanisms one or two *in vivo*. In contrast, mechanism three is consistent with the striking ability of Par-3 clusters to retain autonomy while engaging with cytoskeletal networks or adherens junction precursors. There are clearly close associations between Par-3 clusters and these other mesoscale complexes, but perhaps a single definition of a Par-3 network does not exist. Rather, the integration of Par-3 clusters across a cellular domain seems to depend on various separate networks with specific networks dominating in particular contexts.

What is the best analogy for Par-3 clusters? It can be useful to think of cytoskeletal polymers as ropes or poles, or adhesion complexes as Velcro patches. What everyday material can give us a sense of what Par-3 clusters do? Like caulking, Par-3 clusters can connect various components together into useful conglomerates. Like mucous, Par-3 clusters can sequester factors to prevent their activity elsewhere. Similar to either material, Par-3 clusters don’t move much on their own, are positioned by outside mechanisms, and tend to stay put once moved. Caulking and mucous are not very exciting, but Par-3 clusters have added properties including their continual turnover and chemical regulation, as well as the specificity of their molecular interactions conveyed by the binding pockets of their PDZ domains and other sites. A dynamic and chemically regulated caulking coated with specific binding pockets is not something I have seen at the hardware store. A material with these properties seems ideal for organizing various complexes across large regions of a cell. It could also be useful for nanotechnology.

## Note added in proof

As this review was finalized, three highly significant papers were published by the labs of Goehring ^56^, Goldstein ^57^, and Motegi ^58^. Through various approaches, all three studies demonstrated that Par-3 oligomerization promotes the formation and cortical stabilization of Par-3–Par-6–aPKC clusters, as well as the advective transport of these clusters by the anterior flow of actomyosin that establishes anterior–posterior polarity in the *C. elegans* embryo. By single-molecule pull-down of the complexes from single embryos and the counting of photo-bleaching steps, the Goldstein group demonstrated that the clusters observed *in vivo* are indeed composed of Par protein multimers and that changes to cluster size observed *in vivo* corresponded to the degree of protein multimerization. Each group found that the cluster sizes were maximal as the actomyosin flow established polarity. The Motegi paper demonstrated that cortical tension enhances the clustering of Par-3. Data from both the Goehring and the Motegi studies showed that the Par-3 clusters and separate Cdc-42-enriched plasma membrane domains compete for the Par-6–aPKC complexes, and with a synthetic biology approach the Goehring group found evidence for Par-6–aPKC being inhibited by Par-3 and activated by Cdc-42. Together, these studies suggest that actomyosin contraction increases Par-3 clustering which, in turn, physically enhances the advective transport of Par-3 together with inhibited Par-6–aPKC. The subsequent release of Par-6–aPKC from the Par-3 clusters seems to allow Par-6–aPKC association with Cdc-42 domains, in which Par-6–aPKC has greater activity and a more dispersed distribution for the control of membrane identity. The Goldstein group demonstrated that Polo-like kinase 1 phosphorylates one or more residues in the oligomerization domain of Par-3 to reduce Par-3 clustering after polarity establishment by the contractile actomyosin flow. This regulated reduction in Par-3 clustering would presumably allow the release and local dispersion of Par-6–aPKC for the maintenance of anterior–posterior polarity in the *C. elegans* embryo.
